# Plant Lectins as Medical Tools against Digestive System Cancers

**DOI:** 10.3390/ijms18071403

**Published:** 2017-07-03

**Authors:** Laura Elena Estrada-Martínez, Ulisses Moreno-Celis, Ricardo Cervantes-Jiménez, Roberto Augusto Ferriz-Martínez, Alejandro Blanco-Labra, Teresa García-Gasca

**Affiliations:** 1 Facultad de Ciencias Naturales, Universidad Autónoma de Querétaro, Santiago de Querétaro 76230, Querétaro, Mexico; laurel_1610@hotmail.com (L.E.E.-M.); ulisses.morenoc@gmail.com (U.M.-C.); ricardocervantesjimenez@gmail.com (R.C.-J.); raffer712701@gmail.com (R.A.F.-M.); 2 Unidad de Bioquímica y Biotecnología de Plantas, CINVESTAV Unidad Irapuato, Irapuato 36821, Guanajuato, Mexico; alejandroblancolabra@gmail.com

**Keywords:** cancer, diagnosis tools, digestive system, plant lectins, therapeutic tools

## Abstract

Digestive system cancers—those of the esophagus, stomach, small intestine, colon-rectum, liver, and pancreas—are highly related to genetics and lifestyle. Most are considered highly mortal due to the frequency of late diagnosis, usually in advanced stages, caused by the absence of symptoms or masked by other pathologies. Different tools are being investigated in the search of a more precise diagnosis and treatment. Plant lectins have been studied because of their ability to recognize and bind to carbohydrates, exerting a variety of biological activities on animal cells, including anticancer activities. The present report integrates existing information on the activity of plant lectins on various types of digestive system cancers, and surveys the current state of research into their properties for diagnosis and selective treatment.

## 1. Introduction

Cancer is a complex process in which genetic alterations modify the ability of cells to transduce signals, and allow them to acquire new functions, replicate beyond normal limits, evade apoptosis, and ultimately encroach other tissues [1,2]. Within this process, cell surface glycosylations play a key role on cell development, signalling, interaction, proliferation, differentiation and migration [3,4,5]. Digestive system cancers result from a combination of genetic and lifestyle factors that encompass a wide spectrum of diseases with different clinical characteristics, therapeutic specificities, and life expectancies [6,7]. They represent an important cause of mortality worldwide, generally related to late diagnosis due to the absence of symptoms or masking by other pathologies [8]. Inflammation is a physiological response that has been widely related to the presence of cancer in the digestive tract [9,10], and for which alterations in the glycosylation of proteins play an important role [11]. Although some epidemiological studies have found that from 10 to 15% of cancers were related to infections caused by viruses, fungi or bacteria, it has also been found that up to 25% of cancers are associated with chronic inflammation [12,13,14].

Most of the drugs currently employed in anticancer therapy seem to affect cell replication and therefore tumor growth, but they usually have nonselective mechanisms of action that affect vital macromolecules (such as DNA) or metabolic pathways that are important for both malignant and normal cells, causing undesirable and potentially toxic effects [15,16]. Efforts to treat cancer have led research to focus on the use of less toxic and more selective molecules. Membrane glycosylation, one of the most important facts of cell behaviour, has been pointed to as a valuable target for cancer diagnosis and treatment [17,18]. Cancer cells display aberrant membrane glycosylation patterns, which vary depending on the type of cancer and the tumor stage. Among the major glycosylation changes are the blockage synthesis and the neo-synthesis of carbohydrates, altered branching, and the appearance of new structures. A greater occurrence of cell surface *N*-glycans, sialylations, and fucosylations, the abnormal production of mucin, the expression of Lewis X/A structures in glycosphingolipids (identified as a tumor antigen), and the increased expression of galectins constitute the main structural changes that mark the difference between cancer and normal cells. These changes are related with cell migration, invasion, evasion of immune system, and metastasis [5,19,20,21,22,23].

Lectins are proteins or glycoproteins of non-immune origin that display a ubiquitous distribution in living organisms, and are particularly abundant in plants. They have the ability to recognize and bind specifically and reversibly to either free carbohydrates or glycoconjugates, such as glycoproteins, glycolipids, or polysaccharides, without modifying their structure [24,25,26,27]. This type of proteins has the ability to agglutinate cells or precipitate glycoconjugates detonating a variety of important cellular processes [28,29,30]. Hundreds of plant lectins have been purified and characterized in order to investigate their biochemical properties, carbohydrate binding specificity, and biological functions [31,32], finding numerous applications in the agronomic and biomedical fields, including anticancer potential [31,32,33,34].

## 2. Potential of Plant Lectins against Cancer

The anticancer potential of lectins can be considered from two main angles: diagnostic and therapeutic. The first is due to their ability to recognize cancer cells, mainly by the presence of tumor glycosylations [35,36], which allows for a better diagnosis and prognosis of cancer tumors [37,38]. Their therapeutic potential is based on their antitumor activity and cytotoxic effects through the induction of programmed cell death, such as apoptosis and autophagy [27,39,40,41,42,43,44]; however, the mechanisms of cell death induction have not been fully unravelled [31].

In vitro studies have found a preferential attachment of some lectins to the membranes of cancer cells [27,39,45,46,47], a relevant aspect since selectivity is sought as a tool to improve the effectiveness of anticancer therapies. For example, mistletoe lectins (*Viscum album*) have been used on the European continent for years as alternative adjuvant agents in cancer therapy, lessening the adverse effects of chemo and radiotherapy, and improving patients’ quality of life [47]. Further, some lectins have the ability to bind to the gastrointestinal epithelium cells, exhibiting high resistance to intestinal proteolysis and maintaining their biological activity and carbohydrate affinity intact [48,49]. Table 1 shows the growing diversity of plant lectins with cytotoxic, antiproliferative, apoptotic, or autophagic effects on cancer cell lines and on in vivo experiments.

## 3. Plant Lectins against Esophageal Cancer

Esophageal cancer ranks eighth in prevalence and sixth in cancer mortality worldwide [87], and its incidence is expected to rise within the next few years [88] due to factors such as diet [89,90] and lifestyle that lead to pathologies such as obesity [91], gastresophageal reflux, and Barrett’s esophagus [92,93]—themselves risk factors for changes at the cellular level.

Invasive esophageal cancer is a progressive multi-stage process that can be completed in two ways: by the conversion of normal epithelium to basal cell hyperplasia, dysplasia or carcinoma in situ that leads to squamous cell carcinoma; or by metaplasia caused by Barrett’s esophagus, which represents a previous stage and leads to esophageal adenocarcinoma (EAC) [92,94]. However, the elasticity of the esophagus delays the presence of symptoms [95], so that cancer in this organ is habitually diagnosed in advanced stages and often in the presence of metastatic disease [96], decreasing the 5-year survival to less than 15% [97]. Diagnosis is usually invasive and exhibits limitations in the detection of cancer in its early stages [98]. According to this, and given the fact that esophageal cancer is mostly preceded by dysplastic and metaplastic changes in tissue, it is important to find biomarkers that allow identification of alterations at the cellular level, in the early stages of neoplastic formation [96].

At present, there is little evidence on the use of plant lectins as diagnostic agents or adjuvant treatment of esophageal cancer. However, in a recent study, the topical application of fluorescently labelled wheat germ lectins (WGA) on ex vivo esophagus tissues showed high affinity and specificity for sub-expressed glycans in neoplasia originated from Barrett’s esophagus. Identification of dysplasia was better traced than by white light endoscopy, the technique commonly used for diagnosis [99], confirming that the use of lectins for optically detecting changes in glycan expression in dysplastic tissue represents a potential biomarker for the transformation towards EAC [100]. Additionally, new lectin-based biomarkers have been used for the detection of EAC in serum. A lectin-coated magnetic bead array, coupled with mass spectrometry and assembled with 20 lectins, mostly from plants, has distinguished between healthy, Barrett’s esophagus, and EAC phenotypes and will be subject to further testing [98].

## 4. Plant Lectins against Gastric Cancer

Gastric cancer (GC) is one of the most aggressive malignancies, occupying the fourth place in morbidity and the second in mortality among cancers worldwide [101,102]. In spite of a downward trend in incidence and mortality shown in several countries [103,104], it continues to be a threat in developing countries [101]. This type of cancer tends to progress from chronic gastritis [105,106,107], developing over the course of years and even decades, remaining clinically undetectable in the absence of specific symptoms [108,109,110]. Since it is fatal in about 80% of cases [111] due to diagnosis in advanced stages or even metastasis [110], surgical and chemotherapeutic treatments no longer have the desired effect [112]. Therefore, it is necessary to find more precise markers that allow more efficient diagnosis in the early stages [113].

Gastric cancer shows an outstanding aspect within its multifactorial aetiology, which is the presence of *Helicobacter pylori* bacteria in up to 95% of cases [114]. This bacterium, classified as a class I carcinogen since 1994 [115], has the ability to adhere to epithelial cells and the gastric mucosa by adhesins, extra-membrane proteins that bind to glycosylated receptors from the host, modifying the glycophenotype and promoting infection and chronic inflammation. The resulting alterations in the glycosylations affect the activity of cadherins and integrins, proteins that regulate cell–cell and cell–extracellular matrix interactions, respectively. Proliferation, migration and invasion processes are affected, facilitating carcinogenesis, and therefore representing important targets in anti-cancer therapy [116].

The studies that have been carried out using lectins as tools for GC have been focused on diagnosis due to the differential assessment of healthy and neoplastic tissues, metastasis, or by evaluating recurrence through the analysis of glycans present in different tissues. Lectin microarrays have been used to differentiate between gastric ulcer (GU) and GC. For instance, 40 human GU and GC tissue samples previously diagnosed by pathologists were analysed by a microarray made up by 37 lectins, mostly from plants. Differences between the two diseases were found in the glycopatterns, as well as a higher presence of glycosylations in GC than in GU, which showed higher binding affinity for MPL (*Maclura pomifera*) and VVA (*Vicia villosa*) lectins [117]. Another diagnostically oriented study evaluated the ability of a microarray of 17 lectins integrated in a microfluidic “lab-on-a-chip” platform to identify alterations in the glycan structure of biopsies and serum from 39 patients, either healthy gastric epithelium, type B chronic gastritis associated with *H. pylori*, type C chronic gastritis, or gastric adenocarcinoma. The microarray was able to discriminate between the four clinical stages from tissue samples. For the serum samples, it was only possible to distinguish between normality and disease. Additionally, it was possible to determine the glycoprofiles of the three disease stages [113]. In another study, the expression of glycans was analyzed through a microarray made up of 45 lectins in 60 healthy tissues as well as in 60 tissues resected from gastric cancer patients. Twenty-four out of the 45 lectins tested showed significant differences at binding to cancer tissues in comparison to healthy tissues; in particular, the BPL lectin (*Bauhinia purpurea*) showed promise as predictor of gastric cancer recurrence [118]. Moreover, a microarray constituted of 41 lectins, mostly from plants, was successfully used to differentiate cancer phenotypes through glycan profiling of 242 advanced GC tissue samples, and was found to be a more accurate quantitative assessment than immunostaining. Lymph-node-metastasis-associated lectins were also discerned [119].

On the other hand, recent studies have shifted to analysing the cytotoxic effect of lectins in gastric cancer as a therapeutic possibility. *Pseudomonas fluorescens* lectins (PFL) showed cytotoxicity against human gastric cancer cells (MKN28). A dose-dependent effect on cell viability was shown in doses of 0.5 μM and higher; however, at lower doses a slight increase in cell viability was observed [120]. The cytotoxic and apoptotic effect of *Urtica dioica* (UDA) lectins on human gastric cancer cells (AGS) was likewise tested. The cells were exposed to different concentrations of the lectin for 24 h and a decrease in cell proliferation and apoptosis induction was observed [80].

## 5. Plant Lectins against Small Intestine Cancer

Although the small intestine makes up 75% of the gastrointestinal tract and 90% of the total mucosal surface, the presence of tumors in this organ is rare [121,122], representing only 3% of malignancies [123]. This situation can be explained by factors such as rapid transit, the content of circulating fluid, the presence of the enzyme benzopyrene hydroxylase and low bacterial load, which means less exposure to carcinogens and irritants and less formation of carcinogenic metabolites [124]. However, small intestine cancer prevalence is increasing [122], and like other neoplasias of the gastrointestinal tract lacks specific symptoms until later stages [125], hindering diagnosis and appropriate treatment and reducing lifespan.

Food lectins affect intestinal function since they interact with the small intestine epithelial cells [126] and can remain active for several hours, as they can resist the digestive process. Some of them show tolerance to variables such as elevated temperatures, acid pH, and digestive enzymes [19,127,128]. The administration of raw leguminous beans or their lectins to rats can provoke effects ranging from weight loss to death. Chronic exposure to lectins can cause small intestine hyperplasia [129,130,131]. However, a recent study found that after the administration of *Phaseolus vulgaris* L. var. Beldia to rats, some marked structural changes in the small intestine villi were observed, but not weight loss [132]. The evidence described suggests that the feasibility of lectins for the diagnosis or treatment of small bowel cancer, although no studies have been found on that matter.

## 6. Plant Lectins against Colorectal Cancer

Colorectal cancer (CRC) ranks third in incidence among all types of cancer and has a mortality rate of about 50% [133], with a high prevalence in developed countries [134]. Glycosylation alterations are important changes in the inflammation process, ulcerative colitis, Crohn’s disease, precancerous adenomatous polyps, hyperplastic polyps, and colon cancer [135]. They usually occur in *O*-linked mucin-type glycans that start with *N*-acetylgalactosamine (GalNAc), causing the shortening of *O*-glycans. Plant lectins can recognize these changes and interact with various colon cell types. Taking into account that changes in glycosylations have been associated with the presence of CRC [136,137], and that some lectins remain intact through the intestinal tract [19], they have the potential to be used as diagnosis or therapeutic tools.

Diagnosis of CRC can take advantage of lectin’s recognition properties. A lectin glycoarray was used to detect biomarkers that could discriminate between normal, adenoma, and CRC in human plasma, identified marked differences in CRC and adenomas compared with normal tissues. Changes consisted of a notable elevation of sialylations and fucosylations in complement C3, histidine-rich glycoprotein, and kininogen-1, which were identified as useful biomarkers for this disease [138]. In tissue samples, lectin microarrays have been used for the identification of glycan differences between colon cancer and normal tissues from patients, where *Solanum tuberosum* lectin (STL) recognized with high affinity GlcNAcylation, enabling distinction between both types of tissues [139].

Regarding the cytotoxic effects of plant lectins on CRC, in vitro studies have shown that Korean mistletoe lectins (VCA) exhibit a dose-dependent effect on a cell line of colon cancer (COLO). Approximately 65% of the treated cells showed apoptosis mediated by the activation of caspases-2, -3, -8, and -9 and the inhibition of antiapoptotic proteins. COLO cells were inoculated into naked CD1 nu/nu mice and VCA lectins were injected around the tumor mass for 5 weeks. Complete tumor regression was observed [140]. Lectins from leaves of *Morus alba* (MLL) exhibited cytotoxic effect on HCT-15 cells from human colorectal adenocarcinoma and an antiproliferative effect by apoptosis induction [66]. Additionally, a lectin obtained from *Lotus corniculatus* (LCL) showed a dose-dependent antiproliferative effect on HCT116 cells from human colonic carcinoma by apoptosis induction [141].

Lectins from Tepary beans (*Phaseolus acutifolius*, TBL) exerted a dose-dependent antiproliferative effect on different cancer cell lines [39,67], particularly on human colorectal adenocarcinoma CaCo-2 [39]. In vivo studies showed low TBL toxicity in short-term experiments, depending on the administration route [48,142]. In a study to determine the effect of TBL on colon cancer in rats, a 6-week intragastric administration demonstrated good tolerability with no toxic effects however; a 10% decrement of body weight gain was observed [48]. Similarly, an aqueous lectin extract of *Moringa oleifera* seeds caused moderate cytotoxicity on HT-29 colon cancer cells. When administered to mice in a dose of 2000 mg/kg, no signs of acute or systemic toxicity were observed [143].

However, some lectins exhibit contrary effects on cell proliferation. Peanut agglutinin lectin (PNA) showed a mitogenic effect on HT-29 and SW-1222 cells [144]. This lectin binds to the Thomsen–Friedenreich (TF) oncofetal carbohydrate antigen that is abundant in colon cancer, adenomas, and inflammatory bowel disease, and has shown a mitogenic effect on colon epithelial cells, both in vitro and in vivo. This effect has been related to the mitogen-activated protein kinase (MAPK) pathway [145]. Colon cancer metastasis is also related to signalling pathways such as MAPK [146], ribosomal s6 kinase (RSK) via the extracellular signal-regulated kinase (ERK) [147], proto-oncogene tyrosine-protein kinase (Src) [148], and protein kinase B (Akt) [149,150]. To date, the T-LAK-cell-originated protein kinase (TOPK) pathway has been described as a regulator of the metastasis process in colon cancer cells [150]. Therefore, efforts must focus on molecular targets of signalling pathways to understand the specific effects of lectins on colon cancer.

In vivo studies have shown that some lectins can affect several relevant cellular processes in colon cancer. Chinese mistletoe lectins (ACML-55) induced antitumor immunity in mice inoculated with CT26 colon cancer cells, delaying tumor development [151].

## 7. Plant Lectins against Liver Cancer

Liver cancer is one of the most aggressive cancers, with a high mortality rate worldwide [152]. Its prevalence is higher in Asia and Africa [153], where China accounts for slightly more than half of all deaths worldwide [154]. Infections by hepatitis B and C viruses are the main risk factor for liver cancer, accounting for up to 77% of cases [155]. Other important risk factors are alcoholism, smoking, diabetes mellitus, metabolic syndrome, and exposure to aflatoxins [154,156], all of which are preventable to some extent. Up to 90% of primary liver cancer cases are hepatocellular carcinomas [156].

Of interest for diagnosis, *Lens culinaris* agglutinin (LCA) has been used as a tool for hepatocellular carcinoma identification, taking advantage of its specific binding to α1-6 fucose [157,158]. Research concerning the effect of plant lectins on liver cancer cells has determined that wheat germ lectin (WGA) promotes a high cytotoxic effect [78]. Korean mistletoe lectins (VCA) were tested on the SK-Hep-1 human hepatoma cell line, which expresses p53, and on Hep3B, which does not express p53. A dose- and time-dependent cytotoxic effect was observed on both cell lines, which were similarly affected by both lectins. The study therefore concluded that the mechanism of cell death was independent of p53. Apoptosis induction and inhibition of telomerase were found [83]. In another work, when Hep3B cells were exposed to VCA an apoptotic effect related to the increase of reactive oxygen species and the decreased of mitochondrial membrane potential was reported. The phosphorylation of JNK appeared to be responsible for triggering a modification in the ratio of Bax/Bcl-2, Bax translocation, the consequent release of cytochrome c, and ultimately activation of caspase 3 [159].

Concanavalin A (ConA) lectins inhibit growth and elicit autophagy in ML-14a, Huh-7 and HepG2 hepatoma cell lines. In addition, an in vivo assay injected the spleens of mice with severe combined immunodeficiency with human hepatoma cells, which migrated to the liver to form tumor nodules. One week after inoculation, treatment with intravenous ConA lectin was initiated. The results showed that ConA lectin treatment significantly inhibited the formation of tumor nodules at doses of 20 mg/kg, presumably through lymphocyte activation [60]. *Phaseolus vulgaris* var. blue tiger king (BTKL) lectins were tested on HepG2 cells from human hepatocellular carcinoma and WRL 68 from human embryonic liver tissue. The results suggested a selective cytotoxic effect, affecting HepG2 cells more than their non-carcinogenic counterparts, whose proliferation was not significantly affected. It was also determined that the most prevalent type of cell death was apoptosis, with the presence of DNA fragmentation, apoptotic bodies, chromatin condensation and membrane depolarization; however, necrosis was also found [68]. Lectins of *Phaseolus coccineus* L. var. Albonanus Bailey (CHL) were also tested on HepG2 cells, showing an antiproliferative effect [160].

Recently, the effect of *Momordica Charantia* lectins (MCL) was studied on five human hepatoma cell lines, including HepG2 and PLC/PRF/5. The results showed a dose- and time-dependent cytotoxicity induction that inhibited cell proliferation. The presence of apoptosis and autophagy was specifically detected by G2/M arrest, as well as the activation of MAPK pathways and caspases-3, -8, and -9 in the apoptotic processes. Additionally, in an in vivo xenotransplant-type assay, human hepatoma cells were injected into nude mice, which were then administered with MCL and/or the antineoplastic drug Sorafenib. A dramatic decrease in tumor size by apoptosis was observed in rats treated with the lectin–drug combination. Based on the results, the authors suggest MCL as a promising chemotherapeutic agent [161]. *Allium chinense* lectins (ACL) were studied on Hep-3B human hepatoma cells, where a cytotoxic effect was observed in a dose-dependent manner. Apoptosis by mitochondrial route was determined [54]. Additionally, Broccolini lectin (BL) from *Brassica oleracea* Italica showed a selective dose-dependent cytotoxic effect on HepG2 cells [162].

## 8. Plant Lectins against Pancreatic Cancer

Pancreatic cancer (PC) is one of the most lethal cancers, with a 5-year survival prognosis below 5%, independent of surgical resection of the neoplasm [163,164]. This is because, like most cancers of the digestive tract, is usually diagnosed at advanced stages, commonly when metastasis is already present. This situation is largely due to the inaccessibility of the organ for diagnostic testing, its late clinical presentation, and a lack of biomarkers that identify early pancreatic cancer stages [38,164,165]. Due to its rapid clinical progression, PC remains a real challenge for early detection.

The ability of plant lectins to differentiate between healthy pancreatic tissue and neoplastic tissue, based on their differential affinity to cell glycosylation patterns, has been tested though the use of lectin microarrays. A study was performed on serum samples from 24 patients, both healthy and with confirmed diagnosis of chronic pancreatitis or pancreatic cancer. The samples were processed and subjected to a microarray composed of MAL (*Maackia amurensis*); SNA (*Sambucus nigra*); PNA (*Arachis hypogaea*); ConA (*Canavalia ensiformis*); and a mushroom lectin, AAL (*Aleuria aurentia*), in order to detect differences in glycans. Bioinformatic analyses found that samples from healthy and pancreatitis-affected patients showed greater similarity between them than to samples of pancreatic cancer. The most prominent alterations in the expression of glycosylations during the progression of PC were sialylations and fucosylations in different proteins [165]. In a larger study, a trial was conducted to diagnose structural differences in serum glycans from 183 healthy patients with chronic pancreatitis, type II diabetes mellitus, or pancreatic cancer using an antibody/glycoprotein/lectin sandwich assay with lectins from *Aleuria aurentia* (AAL), *Sambucus nigra* (SNA), *Lens culinaris* (LCA), and *Canavalia ensiformis* (ConA). The results showed that the microarray was able to discriminate between cancer samples, the other pathologies, and the healthy control group with a high sensitivity and specificity, particularly by SNA lectin [166].

In an in vitro study, the effects of wheat germ lectins (WGA), concanavalin A (ConA), and Phytohemagglutinin-L (PHA-L) were tested on membrane binding and proliferation of 9 pancreatic cancer cell lines (BxPC, MIA, Panc-1, CFPAC, ASPC, HS-766T, HTB-147, CaPan-1 and CaPan-2), using a lectin-blot assay and the incorporation of thymidine. A marked dose-dependent cytotoxic effect of WGA lectin was observed on all cancer cell lines, being higher than the effects of the other two lectins, even at a lower concentration. WGA lectin was able to bind to sialic acid residues in membrane glycoproteins, causing chromatin condensation, nucleus fragmentation, and DNA release, and internalization and localization in the cell nucleus were determined [167]. Recently, the activities of *Benincasa hispida* (BhL) and *Datura innoxia* (DiL9) on pancreatic cancer cells lines have been studied. A considerable antiproliferative, dose-dependent effect triggered by a mitochondrial apoptotic pathway, along with anti-angiogenic features, were reported [168].

In vivo experiments showed the effects of mistletoe extracts and lectins from *Viscum album* and were compared to those of Gemcitabine, an antitumor drug used in the treatment of pancreatic cancer. Xenotransplants in athymic nude mice (NMRI nu/nu) were performed using human pancreatic adenocarcinoma cells PAXF 736 and treated with mistletoe extract, mistletoe lectins or Gemcitabine in equivalent doses. Mistletoe extracts showed more antitumor activity than Gemcitabine, presenting partial regressions and total remission of the tumors. Mistletoe lectins showed similar but lower activity than the extract, also with partial regressions [169]. Tröger and colleagues [170] also evaluated the effect of a mistletoe extract on 220 patients with localized advanced cancer or metastatic pancreatic cancer receiving palliative care only (best supportive care-BSC) without chemotherapeutic treatment at the time of the study. The results were favorable, with a clear increase in survival in the 110 patients who received mistletoe extract treatment over the same number of control patients without treatment. In addition, the patients who were given the extract reported a lower presence of adverse events. The authors suggest the administration of such extract as a second line therapy for patients with advanced or metastatic pancreatic cancer. In this phase III study, the effects of mistletoe extract were attributed mostly to the presence of lectins and viscotoxins.

## 9. Lectin-Based Analytical Techniques with Biomedical Applications

Although lectins of the same family are highly conserved in the binding site amino acid residues, the specificity of binding is related with amino acids of different regions of the carbohydrate-binding site. Lectin’s specificity for glycans include mannose and glucose (Man/Glc) for concanavalin A (Con A), *N*-acetylglucosamine (GlcNAc) and Nacetylneuraminic acid (Neu5Ac) for wheat germ agglutinin (WGA), galactose (Gal) and *N*-acetylgalactosamine (GalNAc) for soybean agglutinin (SBA), and Gal for ricin agglutinin (RCA) [171]. The ability of lectins to recognize glycans allows for the use of several analytical methods that take advantage of two important features: specificity and reversible binding. Table 2 shows some of the traditional and modern techniques used for glycans recognition by lectins in biomedicine.

Taking advantage of lectin’s recognition properties, they have also been used for drug delivery. Oral administration is the most conventional method for drug delivery; however, its passage through the gastrointestinal tract entails a series of obstacles such as pH variation, low stability, solubility, bioavailability, and absorption [189]. Lectins’ ability to prevail in aggressive environments (e.g., pH, heat, and enzymes) and interact with cell membrane glycans allows for their use as vehicles for targeted drug delivery [190,191]. Several plant lectins have been used for this purpose and have been aimed toward specific cells and tissues (direct lectin targeting) [192]. An increase in the cellular uptake of lectin-conjugated particles has been reported [193]. Another modality of targeted drug delivery consists of glycans coupled to nanoparticles to target endogenous lectins within specific tissues (reverse lectin targeting) [194,195]. Its use in cancer therapeutics has also been explored, for example, by associating wheat germ agglutinin (WGA) to a paclitaxel-loaded particle, an effective chemotherapeutic for colon cancer. The conjugated molecule was able to exert anti-proliferative activity against colon cancer cell lines Caco-2 and HT-29, showing greater cellular uptake and retention compared to non-conjugated particles [196].

## 10. Final Remarks and Conclusions

Plant lectins as bioactive molecules are characterized by their ability to recognize animal cell carbohydrates. This property enables them to generate cellular responses depending on cell linage, from immune system activation to cancer cell death. Lectins exhibit a vast potential for diagnostic and therapeutic use against cancer due to the cytotoxic, apoptotic, autophagic, and antitumor effects triggered after exposure to these proteins in cells, tissues, and even patients with cancerous processes of the digestive system. The reported information regarding the activity of plant lectins on digestive cancer cell lines indicates the presence of dose- and time-dependent cytotoxicity, generally affected by induction to apoptosis. In vivo experiments have shown inhibition of tumor growth and in some cases even complete remission of tumors. Phase III studies of the effect of plant lectins in cancer patients have shown favorable effects. The ability to induce cell death in a selective manner is a desirable attribute in anticancer therapy and, paradoxically, a trait most of the current chemotherapeutics lack but which lectins have shown. Hence, the growing interest in the study of the activity of plant lectins is due to the biological effects they exert on cancer cells, from identification of tumors to antitumor activity and, additionally, decreased side effects caused by chemotherapeutics. The diagnostic potential of plant lectins has been exposed using microarrays however; despite their multiple beneficial effects, it is important to acknowledge their possible toxicity that depends on the lectin source, the dose, and the administration route. There is a need to increase the study about the biological effects of lectins, and deepen into their molecular mechanisms in order to take advantage of the biomedical potential of these amazing proteins.

## Figures and Tables

**Table 1:** Antineoplastic activity of plant lectins.

Vegetal Source	Lectin	In Vitro Activity	In Vivo Activity	References
*Abrus precatorius*	AGG	Inhibition of protein synthesis, apoptosis induction.	Inhibition of tumor growth and angiogenesis, apoptosis induction.	[50,51,52,53]
*Allium chinense*	ACL	Antiproliferative effect, apoptosis induction.	Not reported.	[54]
*Arachis hypogaea*	PNA	Antiproliferative effect, apoptosis, and autophagy induction by oxidative stress.	Inhibition of tumor growth, apoptosis and autophagy induction.	[46]
*Astragalus membranaceus*	AML	Antiproliferative effect, apoptosis induction by caspases.	Not reported.	[55,56]
*Canavalia ensiformis*	Con A	Antiproliferative effect, autophagy, and apoptosis induction via caspase–mitochondrial pathway.	Inhibition of tumor growth, inhibition of tumor nodule formation.	[57,58,59,60,61,62]
*Glycine max*	SBL	Antiproliferative effect, apoptosis, and autophagy induction by oxidative stress and DNA damage.	Inhibition of tumor growth, apoptosis, and autophagy induction.	[63]
*Momordica charantia*	MCL	Differential antiproliferative effect, apoptosis induction by caspases.	Inhibition of tumor growth, apoptosis induction.	[64,65]
*Morus alba*	MLL	Apoptosis induction.	Not reported.	[66]
*Phaseolus acutifolius*	TBL	Differential antiproliferative effect, apoptosis induction.	Not reported.	[39,67]
*Phaseolus vulgaris*	PHA	Antiproliferative effect, apoptosis induction by death receptors.	Not reported.	[41,68,69]
*Pinellia ternate*	PTL	Antiproliferative effect, apoptosis induction.	Inhibition of tumor growth.	[70,71]
*Polygonatum cyrtonema*	PCL	Differential antiproliferative effect, autophagy, and apoptosis induction by caspases.	Not reported.	[72,73,74]
*Polygonatum odoratum*	POL	Differential antiproliferative effect, autophagy induction by oxidative stress, and apoptosis induction via caspase–mitochondrial pathway and death receptors.	Not reported.	[42,75,76]
*Sophora flavescens*	SFL	Antiproliferative effect, apoptosis induction by caspases.	Inhibition of tumor growth.	[62,77]
*Triticum vulgaris*	WGA	Differential antiproliferative effect, autophagy induction.	Not reported.	[78,79]
*Urtica dioica*	UDA	Antiproliferative effect, apoptosis induction.	Not reported.	[80]
*Viscum album*	ML	Antiproliferative effect, apoptosis induction.	Inhibition of tumor growth and metastasis, prolonged survival rate.	[81,82,83,84,85,86]

AGG, Abrus agglutinin; ACL, *Allium chinense* lectin; PNA, Peanut agglutinin; AML, *Astragalus membranaceus* lectin; Con A, Concanavalin A lectin; SBL, Soybean lectin; MCL, *Momordica charantia* lectin; MLL, Mulberry leaf lectin; TBL, Tepary bean lectin; PHA, *Phaseolus vulgaris* agglutinin; PTL, *Pinellia ternata* lectin, PCL *Polygonatum cyrtonema* lectin; POL, *Polygonatum odoratum* lectin, SFL, *Sophora flavescens* lectin; WGA, Wheat germ agglutinin; UDA, *Urtica dioica* agglutinin; and ML, Mistletoe lectin.

**Table 2:** Lectin-based analytical techniques for glycan detection (modified from [171]).

Technique	Fundament	References
Cell agglutination	Specific recognition of cell membrane carbohydrates or glycoconjugates.	[28,29,172]
Cytochemical and histochemical assays	Recognition of cell surface carbohydrates or glycoconjugates by labelled lectins or immuno-recognition of lectins.	[157,158,173,174]
Enzyme-linked lectin assay (ELLA)	Marked lectins used for binding to immobilized glycoconjugates.	[175,176,177]
Lectin affinity chromatography (LAC)	Affinity chromatography using immobilized lectins.	[178]
Lectin blotting	Qualitative method for detecting carbohydrates moieties in a western blot-like method.	[179,180]
Crossed affinity immunoelectrophoresis	Based in migration patterns changes of glycosylated proteins in an agarose gel which contain an embedded lectin. A second dimension is needed for detecting of the protein with embedded specific antibody in the gel and a final staining of proteins is required.	[175]
Flow cytometry	Lectins labelled with a fluorophore are used in order to detect cell surface glycoconjugates.	[181,182]
Surface plasmon resonance (SPR)	Immobilized lectins to a glass surface (optical biosensor) and binding to carbohydrates in solution is determined as changes in the refractive index.	[183,184]
Lectin microarrays	A panel of immobilized lectins in a chip is used for glycans recognition.	[4,37,139,166,177]
Antibody-Lectin Sandwich Array (ALSA)	Biomarker glycoprofiling by lectins and glycan-binding antibodies.	[158,185,186]
Electrochemical Impedance Spectroscopy biosensors (EIS)	A label-free biosensor based on the lectin–glycan interaction.	[187,188]
